# Impact of Mental Health Visits on Healthcare Cost in Patients with Diabetes and Comorbid Mental Health Disorders

**DOI:** 10.1371/journal.pone.0103804

**Published:** 2014-08-01

**Authors:** Leonard E. Egede, Mulugeta Gebregziabher, Yumin Zhao, Clara E. Dismuke, Rebekah J. Walker, Kelly J. Hunt, R. Neal Axon

**Affiliations:** 1 Health Equity and Rural Outreach Innovation Center, Ralph H. Johnson Department of Veterans Affairs Medical Center, Charleston, South Carolina, United States of America; 2 Center for Health Disparities Research, Division of General Internal Medicine, Medical University of South Carolina, Charleston, South Carolina, United States of America; 3 Division of Biostatistics & Epidemiology, Medical University of South Carolina, Charleston, South Carolina, United States of America; Institute of Psychiatry, United Kingdom

## Abstract

**Purpose:**

To assess the impact of mental health visits (MHV) on the cost of care for Veterans with diabetes and comorbid mental health conditions.

**Methods:**

A national cohort of 120,852 Veterans with diabetes and at least one mental health diagnosis (i.e., substance abuse, depression or psychoses) in 2002 was followed through 2006. Outcomes were pharmacy, inpatient and outpatient costs in 2012 dollars.

**Results:**

Least-square covariate adjusted estimates from the joint model of total VA costs of the number of MHV using December 31, 2012 value dollars indicate that relative to those with fewer MHV, those with 3+ MHV had the lowest mean inpatient cost ($21,406), but the highest mean outpatient and pharmacy cost ($9,727 and $2,015, respectively). If all Veterans who received zero MHV actually received 3+ MHV, we estimate through simulated scenarios that between $32,272,329 and $181,460,247 in inpatient costs would be saved. However, these savings would be offset by additional expenditures of between $1,166,017,547 and $1,166,224,787 in outpatient costs and between $151,604,683 and $161,439,632 in pharmacy costs.

**Conclusions:**

Among Veterans with diabetes and comorbid mental disorders having three or more mental health visits is associated with marginally decreased inpatient cost, but these potential savings seem to be offset by increased outpatient and pharmacy costs.

## Introduction

Individuals with diabetes are more likely than those without diabetes to have mental health disorders. For example, individuals with diabetes have 60% higher odds of major depressive disorder and 123% higher odds of generalized anxiety disorder [1]. Individuals with diabetes are also more likely to have schizophrenia and bipolar disorder than those without diabetes [2], [3]. Our group has previously examined multiple aspects of the relationship between diabetes and depression [3], [4]. Comorbid depression has been associated with poor glycemic control, decreased medication and diet adherence, increased risk of complications, increased health care costs and decreased health-related quality of life [4]. This may be due to the effect of depression on behavioral pathways or physiological pathways [5]. Less is known about the relationship between diabetes and other mental health disorders, but existing reports document similar impacts on outcomes including glycemic control, diabetic complications, quality of life, disability, and medication adherence [3], [6], [7].

Comorbid diabetes and mental health disorders are also associated with increased healthcare utilization and costs. Overall, the cost of diabetes in the U.S. was estimated at $245 billion in 2012, with the largest component of expenditures attributed to inpatient costs [8]. Healthcare expenditures for individuals with diabetes are, on average, 2.3 times higher than for individuals without diabetes [8]. When considering patients with comorbid depression, costs are significantly higher even after adjustment for the presence of variables such as age, sex, race, insurance, and other chronic medical illnesses [3]–[5]. A recent review of healthcare costs for patients with diabetes and comorbid mental health disorders identified 31 studies, 27 of which focused on depression [9]. Overall, comorbid mental health disorders were associated with increased healthcare costs, however the authors suggested the need for further inquiry into allocation of healthcare resources and costs in comorbid conditions beyond depression [9].

We hypothesized that increased frequency of mental health visits would result in favorable net effects on healthcare costs over time. We also hypothesized that while outpatient and pharmacy costs might increase with mental health visits; there would be a corresponding decrease in inpatient costs associated with overall savings. Therefore, the purpose of this study was to determine whether mental health visits, as defined by visit with a VA clinic stop for a mental health or substance abuse service, have an impact on the cost of care for Veterans with diabetes and comorbid mental health conditions.

## Methods

### Data Source and Sample

A national cohort of U.S. Veterans with type 2 diabetes and at least one mental health diagnosis, was created by linking multiple patient and administrative files from the Veterans Health Administration (VHA) National Patient Care and Pharmacy Benefits Management (PBM) databases. Veterans were first selected into a diabetes cohort who had: 1) type 2 diabetes defined by two or more International Classification of Diseases, Ninth Revision (ICD-9) codes for diabetes (250, 357.2, 362.0, and 366.41) in the previous 24 months (2000 and 2001) and 2) ICD-9 codes for type 2 diabetes from inpatient stays and/or outpatient visits on separate days (excluding codes from lab tests and other non-clinician visits) in 2002, and 3) prescriptions for insulin or oral hypoglycemic agents in 2002 based on a previously validated algorithm [10], [11]. Of the 740,195 Veterans included in the diabetes cohort, 120,852 had at least one ICD-9 diagnosis of substance abuse (305.00, 303.9, 305.1–305.9, 304.0–304.9), depression (262.2, 296.3) or psychosis (295.1–295.3, 295.6, 295.4, 295.4) and comprise the study population for the current analysis. Subjects were followed from 2002 until death, loss to follow-up, or through December 2006. Linking the datasets with scrambled social security numbers, we calculated annual patient-specific VA health care costs through VA Decision Support System (DSS) data, which extracts costs from the VA payroll and general ledger. Costs were then classified as inpatient, outpatient (**including emergency department, primary and non mental health specialty care**), and pharmacy. The study was approved by Medical University of South Carolina Institutional Review Board and Ralph H. Johnson Veteran's Affairs Medical Center Research and Development committee. A HIPAA waiver of consent was obtained in order to access medical records due to the need to merge longitudinal data with identifiable information. Patient records were anonymized and de-identified prior to analysis. Department of Veterans Affairs policy and U.S. federal law prohibit disclosure of Veteran information outside the U.S. Veterans Health Administration. However, investigators interested in obtaining data to replicate our analysis can learn more at the VA Information Resource Center (VIReC) at (http://www.virec.research.va.gov/Index.asp).

### Study Variables

#### Outcome variables

The primary outcome variables were three cost types (pharmacy, inpatient, and outpatient including all emergency department, primary care and non-mental health specialty care) measured in 2006 US dollars from the perspective of the federal payer. We adjusted final costs in to reflect 2012 dollar values using http://www.bls.gov/data/inflation_calculator.htm. DSS cost data was applied to VA encounter codes (diagnosis or procedure). Prescription drug costs were identified from the PBM system and summed from the price per dispensed unit. Costs for each study year were represented in person-years to account for censoring.

#### Covariates

The primary covariate was the number of mental health visits (MHV), as defined by VA clinic stop code for a visit in a mental health or substance abuse service, categorized as 0, 1, 2 and 3+. Clinic stop codes are used by VHA to track clinic workload and productivity, and they represent discrete healthcare encounters. Other covariates included in the full model were age, gender, marital status, service connected disability level, race/ethnicity, residence, region, and comorbidities. Marital status was defined as single or married. Service connected disability level was categorized as <50 or ≥50%. Veterans with high levels of service-connected disability (i.e., > 50%) are exempted from co-payments. Race/ethnicity was classified as non-Hispanic white (NHW), non-Hispanic black (NHB), Hispanic, and other/unknown/missing. Location of residence was defined as urban and rural/highly rural [12], and hospital region was defined by the five geographic regions of the country based on VHA Veteran's Integrated Service Networks (VISNs): Northeast (VISNs 1, 2, & 3), Mid-Atlantic (VISNs 4, 5, 6, 9, & 10), South (VISNs 7, 8, 16, & 17), Midwest (VISNs 11, 12, 15, 19, & 23), and West (VISNs 18, 20, 21, & 22). Comorbidity variables based on ICD-9 codes included, anemia, cancer, cerebrovascular disease, congestive heart failure, cardiovascular disease, hypertension, hypothyroidism, liver disease, lung disease, fluid and electrolyte disorders, obesity, psychoses, and other (AIDS, rheumatoid arthritis, renal failure, peptic ulcer disease and bleeding, weight loss).

### Statistical Analyses

Descriptive measures including mean and median costs were computed for each cost type for each MHV group. Preliminary analysis included plotting the unadjusted mean costs in each cost type (inpatient, outpatient, pharmacy) over time by MHV, with time on the x-axis and unadjusted mean cost on the y-axis. The plots helped to examine trends over time in each source of cost by MHV before adjusting for any other covariates. To model the relationship between the three cost categories and covariates in a manner that accounted for differences in variance among the three outcomes, a joint model based on a multivariate generalized linear mixed model (mGLMM) approach with shared random intercept and slope was used [13].

Since the response variable is a vector of three correlated cost outcomes, mGLMM with a random intercept and slope was used to account for the correlation among the cost outcomes and estimate the joint effect of MHV on the three outcomes. This is implemented in SAS Proc GLIMMIX (SAS Institute Inc, Cary, NC) using SAS code provided in [13]. To account for the skewness in the observed cost data, a log-normal distribution with an identity link was used. Hence the exponent of the parameter estimates can be interpreted as the percent change in each type of cost as a function of unit change in the covariates. Comparison with analysis results based on fitting separate models for each outcome was also performed (see online Table 1). In the joint models, the random intercept shared by the three cost outcomes captures the association in the natural heterogeneity among the individual subjects' inpatient, outpatient and pharmacy costs, while the random slope captures the correlation in the trajectory over time of cost outcomes. Model goodness of fit was assessed using pseudo-AIC-type statistics (based on pseudo-likelihood as implemented in Proc GLIMMIX) and using residual plots.

**Table 1:** Demographic characteristics of the cohort by number of mental health visits (MHV).

*Variable*	*All (n = 120,852)*	MHV = 0* (n = 31,785)	MHV> = 1* (n = 89,067)
Mental Health Care Visits, n, Mean (sd) range			
2002	8.0 (24.7) 0–616		10.8 (28.1) 0–616
2003	5.9 (17.9) 0–424		7.4 (19.8) 0–424
2004	9.3 (29.5) 0–1009		11.9 (32.8) 0–1009
2005	6.5 (20.5) 0–576		8.2 (22.7) 0–576
2006	5.9 (19.4) 0–489		7.4 (21.4) 0–489
Age, years Mean (sd)	61.0 (11.4)	67.0 (10.8)	58.9 (10.9)
Male (%)	96.4	96.9	96.2
Race (%)			
NHW	67.3	77.1	63.8
NHB	15.9	10.5	17.8
Hispanic	6.7	4.0	7.7
Others	10.1	8.3	10.7
Married (%)	53.9	61.0	51.3
Service Connected Disability* † (%)	39.0	25.6	43.7
Rural (%)	37.4	44.2	34.9
Geographic Region (%)			
Mid-Atlantic %	22.2	20.8	22.7
West %	16.8	16.3	17.0
Northeast %	10.4	9.0	10.9
South %	30.1	28.8	30.5
Midwest %	20.0	24.6	18.3
Number of Psychiatric Comorbidities			
One	81.7	93.3	77.5
Two	15.2	6.3	18.4
Three	3.1	0.4	4.0
Psychiatric Comorbidities			
Substance Abuse	22.6	18.0	24.2
Depression	73.3	75.9	72.3
Psychoses	25.6	13.3	30.0
Medical Comorbidities			
Anemia	10.5	13.5	9.4
Cancer	7.1	10.4	5.9
Cerebrovascular disease	16.9	22.1	15.1
Congestive heart failure	13.4	19.7	11.1
Cardiovascular disease	4.2	5.8	3.7
Hypertension	79.0	80.7	78.4
Hypothyroidism	7.2	7.7	7.0
Liver disease	7.0	6.0	7.4
Lung disease	19.6	21.6	18.8
Fluid/electrolyte disorders	9.2	10.6	8.7
Obesity	16.3	13.6	17.3
Other disease	5.6	6.4	5.3
PVD	13.0	17.6	11.3

*Stratified by number of mental health visits in 2002;

† Greater than 0.5 service connected disability.

Potential savings were estimated by examining the adjusted mean cost differential between those with no mental health visits and those with one or more MHV in every cost category in every year and multiplying by the number of Veterans in that year. Finally, projected annual potential total and incremental savings (losses) to the VA at each MHV value was estimated using the 5 year least-square mean estimates (means that are adjusted for covariates as computed by the LSMEANS statement of PROC GLIMMIX) from the joint model. Costs and potential savings were calculated, from the VHA perspective, of providing 3 MHV to individuals with mental health co-morbidities receiving zero MHV were made based on number of Veterans. For the number of Veterans in a MHV category using any service and having any type of cost the high value was used. For those Veterans who had non-negative cost only the low number was used. The U.S. Department of Labor Statistics estimate of the medical component of the Consumer Price Index (CPI) was used to convert all 2002–2005 costs to 2006 dollars prior to model estimation. The CPI was also used to convert potential savings (losses) to the VA from increased mental health visits from 2006 dollars to December 31, 2012 dollar values. All statistical analyses were performed using SAS 9.2 (SAS Institute, Carey, NC).

## Results

### Population Characteristics and Mental Health Visit (MHV) Status

The final study sample consisted of 120,852 Veterans with a diagnosis of type 2 diabetes and either depression, substance abuse, or psychosis during 2002 and followed until death, loss to follow-up or through 2006 (Table 1). The proportion of the cohort with diagnoses of depression, psychoses and substance abuse were 73.3%%, 25.6%% and 22.6%, respectively. Moreover, 81.7% had only one mental health co-morbidity, 15.2% had two mental health co-morbidities and 3.1% had all three mental health co-morbidities. Of the 120,785 Veterans with diagnosed mental health co-morbidity, 26.3% did not have a mental health visit from 2002 through 2006. Among those who had at least one MHV, the mean number of mental health visits declined from 2002 (10.8 visits) to 2003 (7.4 visits) and then reached its peak in 2004 (11.9 visits). Those who had at least one MHV were younger, more likely to be a minority (i.e., NHB, Hispanic or other), more likely to have greater than 50% service connected disability, less likely to be married and have a rural residence, and more likely to have multiple mental health co-morbidities than those without MHV.

### Longitudinal Costs Associations with MHV

Table 2 highlights findings of significant (P<0.001) cost associations with mental health visit (MHV) after adjustment for demographics and the type of medical and mental health co-morbidities and accounting for the correlation of cost categories over time. Relative to the zero MHV group, one MHV was associated with a 1.4% higher inpatient cost, 30.9% higher outpatient cost, and 11.9% higher pharmacy cost between 2002 and 2006. Having two MHV was associated with 5.4% higher inpatient cost, 70.3% higher outpatient cost and 30.8% higher pharmacy cost. Having three mental health co-morbidities was associated with a 4% lower inpatient, 164% higher outpatient, and 71.4% higher pharmacy cost.

**Table 2:** Joint multivariate generalized linear mixed model based on shared random intercept and slope.

	Inpatient Cost		Outpatient Cost		Pharmacy Cost	
Effect	Estimate	95%CI	Estimate	95%CI	Estimate	95%CI
Intercept	14854	(13687, 16121)	3987	(3826, 4155)	1530	(1472, 1590)
Mental Health Visits						
Zero	Reference		Reference		Reference	
One	1.014	(0.984, 1.044)	**1.309**	**(1.295, 1.323)**	**1.119**	**(1.109, 1.130)**
Two	**1.054**	**(1.018, 1.090)**	**1.703**	**(1.681, 1.725)**	**1.308**	**(1.294, 1.323)**
Three or more	**0.960**	**(0.940, 0.980)**	**2.640**	**(2.618, 2.663)**	**1.714**	**(1.701, 1.727)**
Fiscal year	**0.988**	**(0.983, 0.994)**	**0.977**	**(0.975, 0.980)**	**0.978**	**(0.976, 0.980)**
Age	1.001	(1.000, 1.002)	**0.991**	**(0.991, 0.991)**	**0.988**	**(0.988, 0.988)**
Male	**1.094**	**(1.044, 1.147)**	**0.859**	**(0.839, 0.881)**	**0.943**	**(0.922, 0.965)**
Race						
NHW	Reference		Reference		Reference	
NHB	**1.074**	**(1.049, 1.100)**	**1.183**	**(1.167, 1.199)**	**0.845**	**(0.835, 0.856)**
Hispanic	0.973	(0.940, 1.008)	**1.086**	**(1.066, 1.107)**	**0.839**	**(0.825, 0.854)**
Others	**0.942**	**(0.911, 0.973)**	**0.907**	**(0.893, 0.921)**	**0.729**	**(0.718, 0.740)**
Married	**0.784**	**(0.769, 0.798)**	**0.935**	**(0.926, 0.944)**	**1.151**	**(1.140, 1.161)**
Rural	0.993	(0.974, 1.013)	**0.917**	**(0.908, 0.926)**	**1.041**	**(1.032, 1.051)**
Geographic Region						
Mid-Atlantic	Reference		Reference		Reference	
West	**1.081**	**(1.050, 1.112)**	**1.611**	**(1.587, 1.635)**	1.007	(0.993, 1.021)
Northeast	**1.175**	**(1.136, 1.215)**	**1.236**	**(1.215, 1.257)**	**1.059**	**(1.043, 1.076)**
South	**0.908**	**(0.887, 0.930)**	**1.015**	**(1.003, 1.028)**	**1.074**	**(1.061, 1.086)**
Midwest	1.012	(0.985, 1.040)	**1.143**	**(1.127, 1.159)**	**1.099**	**(1.085, 1.114)**
Psychiatric Comorbidities						
Substance Abuse	**1.194**	**(1.167, 1.221)**	0.994	(0.981, 1.007)	**0.734**	**(0.725, 0.743)**
Depression	1.014	(0.992, 1.037)	**1.079**	**(1.065, 1.093)**	**1.175**	**(1.161, 1.190)**
Psychoses	**1.360**	**(1.332, 1.389)**	**1.083**	**(1.070, 1.096)**	**1.302**	**(1.287, 1.317)**
Medical Comorbidities						
Anemia	**1.276**	**(1.243, 1.310)**	**1.322**	**(1.301, 1.343)**	**1.173**	**(1.156, 1.190)**
Cancer	**1.162**	**(1.123, 1.202)**	**1.262**	**(1.239, 1.286)**	**1.158**	**(1.138, 1.178)**
Cerebrovascular disease	**1.247**	**(1.219, 1.275)**	**1.210**	**(1.195, 1.225)**	**1.189**	**(1.175, 1.203)**
Congestive heart failure	**1.165**	**(1.136, 1.194)**	**1.264**	**(1.246, 1.283)**	**1.189**	**(1.173, 1.205)**
Cardiovascular disease	**1.196**	**(1.152, 1.242)**	**1.232**	**(1.204, 1.262)**	**1.053**	**(1.030, 1.076)**
Hypertension	1.020	(0.996, 1.044)	**1.212**	**(1.198, 1.226)**	**1.272**	**(1.258, 1.285)**
Hypothyroidism	1.026	(0.992, 1.061)	**1.059**	**(1.040, 1.078)**	**1.132**	**(1.114, 1.151)**
Liver disease	**1.168**	**(1.134, 1.203)**	**1.214**	**(1.192, 1.237)**	**1.044**	**(1.026, 1.063)**
Lung disease	**1.129**	**(1.106, 1.154)**	**1.221**	**(1.207, 1.236)**	**1.243**	**(1.230, 1.257)**
Fluid/electrolyte disorders	**1.228**	**(1.197, 1.260)**	**1.348**	**(1.326, 1.371)**	**1.079**	**(1.062, 1.096)**
Obesity	**0.968**	**(0.945, 0.992)**	**1.042**	**(1.029, 1.055)**	**1.055**	**(1.043, 1.067)**
Other disease	1.213	(1.174, 1.254)	1.306	(1.280, 1.333)	1.293	(1.269, 1.318)
PVD	1.263	(1.232, 1.295)	1.265	(1.247, 1.283)	1.130	(1.115, 1.145)

### Estimates of Mean and Total VA Costs for Number of MHV

Least-square estimates from the joint model of total VA costs of the number of MHV based on December 31, 2012 value dollars are shown in Table 3. Over the 5 year period, mean inpatient cost was highest for those with two MHV ($23,537) and lowest for those with three or more MHV ($21,406). Mean outpatient cost was highest for those with three or more MHV ($9,727) and lowest for those with zero MHV ($3,631). Mean pharmacy cost was highest for those with three or more MHV ($2,015) and lowest for those with zero MHV ($1,171). Least-square estimates from the joint model of total VA costs of the number of MHV based on December 31, 2012 value dollars by year for 2002 through 2005 are found online (Table S1) which consists of three panels for inpatient (panel a), outpatient (panel b) and pharmacy (panel c) costs.

**Table 3:** Total Cost by number of mental health visits (MHV) over the five year period from Joint Model Least-square Estimates of Inpatient, Outpatient and Pharmacy Cost In December 31, 2012 Value.

			Veterans Impacted (n)		Total Cost ($)	
	#MHV	LSM2012	Low	High	Low	High
Inpatient	0	$ 22,355	34028	191332	$ 760,684,805	$ 4,277,164,248
	1	$ 22,811	9580	46943	$ 218,528,516	$ 1,070,812,541
	2	$ 23,537	6765	30830	$ 159,230,109	$ 725,656,209
	3	$ 21,406	49497	172943	$ 1,059,546,030	$ 3,702,064,145
Outpatient	0	$ 3,631	191298	191332	$ 694,647,222	$ 694,770,683
	1	$ 4,829	46940	46943	$ 226,668,448	$ 226,682,935
	2	$ 6,280	30825	30830	$ 193,584,666	$ 93,616,066
	3	$ 9,727	172926	172943	$ 1,681,969,052	$ 1,682,134,403
Pharmacy	0	$ 1,171	179676	191332	$ 210,362,778	$ 224,009,501
	1	$ 1,317	45622	46943	$ 60,078,270	$ 61,817,856
	2	$ 1,548	30276	30830	$ 46,867,980	$ 47,725,586
	3	$ 2,015	171842	172943	$ 346,185,426	$ 348,403,452

Note: Low number of Veterans impacted is for those Veterans having estimated positive costs in all three cost categories. High number of Veterans impacted is for all Veterans in the sample with a non-zero cost in at least one category. Low Total Cost estimates refer to the low number of Veterans while high Total Cost estimates refer to the high number of Veterans. Mean estimated cost in each category refers to the 5 year least-square mean estimate from the joint model multiplied by the consumer price index multiplier to reflect December 2012 dollar values.

Potential savings to the VA under different possible scenarios of MHV are computed and the results for are shown in Table 4 where total costs were calculated by multiplying the mean least-square estimate from the joint model by the number of Veterans in the low and high categories. The mean least-square estimate was multiplied by the consumer price index multiplier to approximate December 2012 dollar values. We considered two scenarios based on number of Veterans. For the number of Veterans in a MHV category using any service and having any type of estimated cost the high value was used. For those Veterans who had estimated cost only, the low number was used. If all Veterans who received zero MHV, actually received three or more MHV, we estimated that between $32,272,329 and $181,460,247 in inpatient costs would be saved. However, these savings would be offset by additional expenditures of between $1,166,017,547 and $1,166,224,787 in outpatient costs and between $151,604,683 and $161,439,632 in pharmacy costs.

**Table 4:** Estimated VA Savings (Losses) From 0 MHV Veterans Receiving 3 or more MHV in December 31, 2012 Value.

	Veterans Impacted (n)		Mean Cost ($)		Estimated Savings (Losses)	
	Low	High	0 MHV	3+MHV	Low	High
Inpatient	34028	191332	$ 22,355	$ 21,406	$ 32,272,329	$ 181,460,247
Outpatient	191298	191332	$ 3,631	$ 9,727	$ (1,166,017,547)	$ (1,166,224,787)
Pharmacy	179676	191332	$ 1,171	$ 2,015	$ (151,604,683)	$ (161,439,632)

Note: Low number of Veterans impacted refers to the number of Veterans with estimated positive costs in all three categories. High number of Veterans impacted refers to the total number of Veterans in the sample with a non-zero cost in at least one category. Low estimated Savings (Losses) refer to the low number of Veterans impacted while high estimated Savings (Losses ) refer to the high number of Veterans. Mean estimated cost in each category refers to the 5 year least-square mean estimate from the joint model multiplied by the consumer price index multiplier to reflect December 2012 dollar values.

## Discussion

Our research team has previously examined the effects of medication non-adherence on costs and the potential cost savings from improving adherence [13]. We found that improving diabetic medication adherence would result in annual estimated cost savings ranging from ∼$661 million to ∼$1.16 billion. Using similar methods, we hypothesized that increased frequency of mental health visits would result in favorable net effects on healthcare costs over time. As anticipated, inpatient costs were lowest in Veterans with diabetes with mental health comorbidities who had three or more MHV, even after adjusting for demographic factors, psychiatric comorbidities, and medical comorbidities. Outpatient and pharmacy costs were highest in this group as well. However, contrary to our hypothesis, the dollars saved on inpatient costs were not sufficient to offset the increased outpatient and pharmacy cost in Veterans with three or more MHV relative to those with zero, one, or two MHV. Potential savings of providing 3 MHV to Veterans with mental health comorbidities currently receiving zero MHV indicate the VA would save between $32,373,329 and $181,460,247 on inpatient cost. However, this savings would be offset by additional expenditures of between $1,166,017,547 and $1,166,224,787 in outpatient costs and between $151,604,683 and $161,439,632 in pharmacy costs.

On August 31, 2012, President Barack Obama signed an executive order designed to improve access to mental health services for Veterans, service members, and military families [14]. This order contained provisions to improve suicide prevention programs, enhance partnerships between the Department of Veterans Affairs and community providers, improve mental health research and development, and expand VA mental health services staffing. Specifically, the VA is currently in the process of hiring over 1,600 additional mental health providers and 800 new peer counselors. This analysis is relevant as one approach to answer questions regarding how to accomplish these goals, especially as it pertains to high-risk patients with comorbid medical illnesses like diabetes. In particular, how should VA mental health providers allocate their time and resources given a contemporary environment of belt-tightening and constricting federal budgets?

Multiple studies have documented that, among patients with comorbid diabetes and psychiatric disorders, poor disease control in one domain can adversely impact disease control and outcomes in the other [15]. Several studies also suggest that increased mental health participation can lead to improvements in diabetes control. For example, Dixon et al. analyzed diabetes control in patients with comorbid schizophrenia and severe mood disorders [16]. They found lower hemoglobin A1C values among patients actively enrolled in mental health treatment compared to individuals with diabetes without mental health disorders and concluded that there may be previously unrecognized benefits for diabetes management among persons with severe mental illnesses who receive regular mental health care. Similarly, Kreyenbuhl et al. found better adherence in patients with schizophrenia and diabetes than patients with only diabetes, and they suggested enhanced awareness to overall medical well being as an outcome of mental health treatment [17]. They also found a greater number of visits increased the odds of receiving ACE-inhibitors to reduce cardiovascular risk [18]. While these studies indicate that high-quality mental health care may contribute to improved diabetes control, our analysis raises concerns that increasing clinic visits alone may not be the best approach for reducing utilization and cost.

Because inpatient cost reduction tends to drive cost savings, it is likely that a more nuanced approach to the provision of mental health services is required, one that tailors service provision to individual patient needs, severity of illness, and local resources. For example, patients with diabetes and mild depression may not benefit from multiple mental health visits as much as patients with more severe depression, substance abuse, or schizophrenia. There may also be other care models besides mental health clinic visits that better suit the needs of some Veterans. The VHA has led innovation in developing models for co-locating primary care and mental health services [19]–[22]. The VHA has also developed leading telemental health programs, which increase access to care for mental health services for many Veterans, especially in rural areas [21], [23]–[26]. Finally, Veterans with the highest severity of mental illness will likely benefit from intensive case management strategies, the cost outlays for which are not likely captured by current workload models [27].

This study should be interpreted in light of certain limitations. First, only 3.6% of our sample was female which may limit the generalizability of our findings for women Veterans. However, our cohort did contain over 4,000 women. Second, our database did not contain additional information regarding potential cost predictors such as self-care behaviors, disease knowledge, and health beliefs. Unfortunately, collecting such information was not feasible for such a large cohort. Our study lacked cost data from other payers such as Medicare. Thus, our cost estimates may be low if subjects tended to use large amounts of non-VHA care. However, our inclusion criteria tended to select patients who utilize VHA for most of their healthcare. In addition, since our focus was on multimorbidity, we chose to analyze the total number of mental health visits as a count rather than examining number of visits by individual mental health comorbidity. Future estimates of cost savings should explore the impact of discrete mental health disorders on cost and outcomes in this population. Finally, future studies are needed that incorporate adjustment for severity of illness within mental health disease categories and that stratify costs by clinical outcomes. In summary, patients with diabetes and comorbid mental health disorders represent a high-risk patient population that requires extensive resource allocation to improve health outcomes. We expect that these results will be of interest to healthcare providers and policy makers who strive to achieve optimal outcomes for patients while managing costs in a national healthcare system. First, providers must develop and select healthcare treatments and programs among the menu of available alternatives. Our results suggest that a simplistic approach that simply increases provider capacity and visit numbers may not lead to improvement in inpatient healthcare utilization substantial enough to offset outpatient and pharmacy investments. Instead, other evidence-based strategies mentioned above e.g., co-located primary care, telemental health, intensive case management) may be preferred. In turn, healthcare executives can use modeling techniques as described here in order to better plan for the financial impact of programs selected for implementation at the local and regional level. Finally, national healthcare payors such as Department of Veterans Affairs or Medicare can utilize this type of analysis in long-range budget planning.

## Supporting Information

Table S1 **Panel A.** Inpatient Total Cost by number of mental health visits (MHV) by year from Joint Model Least-square Estimates of Inpatient, Outpatient and Pharmacy Cost In December 31, 2012 Value **Panel B.** Outpatient Total Cost by number of mental health visits (MHV) by year from Joint Model Least-square Estimates of Inpatient, Outpatient and Pharmacy Cost In December 31, 2012 Value. **Panel C.** Pharmacy Total Cost by number of mental health visits (MHV) by year from Joint Model Least-square Estimates of Inpatient, Outpatient and Pharmacy Cost In December 31, 2012 Value.
(DOCX)
