# Supplementary material for: Impact of Mental Health Visits on Healthcare Cost in Patients with Diabetes and Comorbid Mental Health Disorders
Source: PLoS One. 2014 Aug 1;9(8):e103804. doi: 10.1371/journal.pone.0103804 (PMC4118960; doi:10.1371/journal.pone.0103804)
Supplement: Table S1 — Panel A. Inpatient Total Cost by number of mental health visits (MHV) by year from Joint Model Least-square Estimates of Inpatient, Outpatient and Pharmacy Cost In December 31, 2012 Value Panel B. Outpatient Total Cost by number of mental health visits (MHV) by year from Joint Model Least-square Estimates of Inpatient, Outpatient and Pharmacy Cost In December 31, 2012 Value. Panel C. Pharmacy Total Cost by number of mental health visits (MHV) by year from Joint Model Least-square Estimates of Inpatient, Outpatient and Pharmacy Cost In December 31, 2012 Value. (DOCX) [file pone.0103804.s001.docx]

**ONLINE MATERIAL**

Table S1 Panel A. Inpatient Total Cost by number of mental health visits (MHV) by year from Joint Model Least-square Estimates of Inpatient, Outpatient and Pharmacy Cost In December 31, 2012 Value

|  | |  | |  | | Veterans Impacted (n) | | | | Total Cost ($) | | | |
| --- | --- | --- | --- | --- | --- | --- | --- | --- | --- | --- | --- | --- | --- |
| #MHV | | Year | | LSM2012 | | Low | | High | | Low | | High | |
| 0 | | 2002 | | $ 22,029 | | 10122 | | 51660 | | $ 222,973,905 | | $ 1,137,999,597 | |
| 0 | | 2003 | | $ 22,631 | | 2936 | | 16235 | | $ 66,445,967 | | $ 367,421,758 | |
| 0 | | 2004 | | $ 23,286 | | 6700 | | 39070 | | $ 156,018,456 | | $ 909,797,174 | |
| 0 | | 2005 | | $ 22,001 | | 7322 | | 43038 | | $ 161,091,496 | | $ 946,880,062 | |
| 0 | | 2006 | | $ 21,857 | | 6948 | | 41329 | | $ 151,864,759 | | $ 903,341,771 | |
| 1 | | 2002 | | $ 22,208 | | 2925 | | 12007 | | $ 64,959,531 | | $ 266,656,099 | |
| 1 | | 2003 | | $ 23,220 | | 829 | | 4141 | | $ 19,249,284 | | $ 96,153,541 | |
| 1 | | 2004 | | $ 24,148 | | 1653 | | 8431 | | $ 39,916,504 | | $ 203,591,075 | |
| 1 | | 2005 | | $ 23,077 | | 2145 | | 11638 | | $ 49,500,197 | | $ 268,570,300 | |
| 1 | | 2006 | | $ 21,492 | | 2028 | | 10726 | | $ 43,585,731 | | $ 230,522,955 | |
| 2 | | 2002 | | $ 22,762 | | 2005 | | 7915 | | $ 45,636,820 | | $ 180,157,322 | |
| 2 | | 2003 | | $ 23,028 | | 579 | | 2722 | | $ 13,333,336 | | $ 62,682,801 | |
| 2 | | 2004 | | $ 24,670 | | 1463 | | 7081 | | $ 36,092,578 | | $ 174,690,052 | |
| 2 | | 2005 | | $ 23,984 | | 1425 | | 6559 | | $ 34,177,200 | | $ 157,311,055 | |
| 2 | | 2006 | | $ 23,293 | | 1293 | | 6553 | | $ 30,118,186 | | $ 152,640,739 | |
| 3 | | 2002 | | $ 22,106 | | 16731 | | 50883 | | $ 369,854,212 | | $ 1,124,815,722 | |
| 3 | | 2003 | | $ 21,999 | | 3909 | | 14181 | | $ 85,995,102 | | $ 311,971,486 | |
| 3 | | 2004 | | $ 21,992 | | 10454 | | 39480 | | $ 229,905,958 | | $ 868,250,165 | |
| 3 | | 2005 | | $ 21,021 | | 9924 | | 36926 | | $ 208,608,036 | | $ 776,205,195 | |
| 3 | | 2006 | | $ 19,993 | | 8479 | | 31473 | | $ 169,520,104 | | $ 629,237,673 | |
|  |  | |  | |  | |  | |  | |  | |  |
|  |  | |  | |  | |  | |  | |  | |  |

|  |  |  |  |  |  |  |
| --- | --- | --- | --- | --- | --- | --- |

Table S1, Panel B. Outpatient Total Cost by number of mental health visits (MHV) by year from Joint Model Least-square Estimates of Inpatient, Outpatient and Pharmacy Cost In December 31, 2012 Value

|  | |  |  | Veterans Impacted (n) | | Total Cost ($) | |
| --- | --- | --- | --- | --- | --- | --- | --- |
| #MHV | | Year | LSM2012 | Low | High | Low | High |
| 0 | 2002 | $ 3,984 | 51655 | 51660 | $ 205,813,676 | $ 205,833,598 |  |
| 0 | 2003 | $ 3,146 | 16234 | 16235 | $ 51,065,485 | $ 51,068,631 |  |
| 0 | 2004 | $ 4,331 | 39056 | 39070 | $ 169,143,125 | $ 169,203,756 |  |
| 0 | 2005 | $ 3,394 | 43028 | 43038 | $ 146,019,213 | $ 146,053,149 |  |
| 0 | 2006 | $ 3,428 | 41325 | 41329 | $ 141,641,950 | $ 141,655,660 |  |
| 1 | 2002 | $ 5,116 | 12005 | 12007 | $ 61,413,515 | $ 61,423,747 |  |
| 1 | 2003 | $ 4,478 | 4141 | 4141 | $ 18,543,747 | $ 18,543,747 |  |
| 1 | 2004 | $ 5,103 | 8431 | 8431 | $ 43,026,827 | $ 43,026,827 |  |
| 1 | 2005 | $ 4,651 | 11637 | 11638 | $ 54,124,975 | $ 54,129,626 |  |
| 1 | 2006 | $ 4,829 | 10726 | 10726 | $ 51,792,556 | $ 51,792,556 |  |
| 2 | 2002 | $ 6,050 | 7912 | 7915 | $ 47,870,046 | $ 47,888,197 |  |
| 2 | 2003 | $ 5,861 | 2722 | 2722 | $ 15,952,673 | $ 15,952,673 |  |
| 2 | 2004 | $ 7,470 | 7081 | 7081 | $ 52,896,168 | $ 52,896,168 |  |
| 2 | 2005 | $ 6,049 | 6558 | 6559 | $ 39,668,293 | $ 39,674,342 |  |
| 2 | 2006 | $ 6,097 | 6552 | 6553 | $ 39,947,335 | $ 39,953,432 |  |
| 3 | 2002 | $ 9,771 | 50877 | 50883 | $ 497,141,625 | $ 497,200,254 |  |
| 3 | 2003 | $ 9,229 | 14181 | 14181 | $ 130,874,249 | $ 130,874,249 |  |
| 3 | 2004 | $ 11,667 | 39477 | 39480 | $ 460,563,729 | $ 460,598,729 |  |
| 3 | 2005 | $ 9,003 | 36920 | 36926 | $ 332,402,892 | $ 332,456,912 |  |
| 3 | 2006 | $ 9,190 | 31471 | 31473 | $ 289,231,345 | $ 289,249,726 |  |

Table S1, Panel C. Pharmacy Total Cost by number of mental health visits (MHV) by year from Joint Model Least-square Estimates of Inpatient, Outpatient and Pharmacy Cost In December 31, 2012 Value

|  |  |  | Veterans Impacted (n) | | Total Cost ($) | |
| --- | --- | --- | --- | --- | --- | --- |
| #MHV | Year | LSM2012 | Low | High | Low | High |
| 0 | 2002 | $ 1,258 | 51655 | 51660 | $ 64,983,916 | $ 64,990,206 |
| 0 | 2003 | $ 1,203 | 15061 | 16235 | $ 18,124,332 | $ 19,537,117 |
| 0 | 2004 | $ 1,220 | 36020 | 39070 | $ 43,953,817 | $ 47,675,614 |
| 0 | 2005 | $ 1,131 | 39122 | 43038 | $ 44,232,642 | $ 48,660,203 |
| 0 | 2006 | $ 1,053 | 37818 | 41329 | $ 39,831,310 | $ 43,529,224 |
| 1 | 2002 | $ 1,281 | 12004 | 12007 | $ 15,381,868 | $ 15,385,712 |
| 1 | 2003 | $ 1,297 | 3972 | 4141 | $ 5,152,669 | $ 5,371,904 |
| 1 | 2004 | $ 1,364 | 8024 | 8431 | $ 10,945,987 | $ 11,501,198 |
| 1 | 2005 | $ 1,351 | 11236 | 11638 | $ 15,181,139 | $ 15,724,288 |
| 1 | 2006 | $ 1,293 | 10386 | 10726 | $ 13,424,842 | $ 13,864,322 |
| 2 | 2002 | $ 1,502 | 7913 | 7915 | $ 11,885,440 | $ 11,888,444 |
| 2 | 2003 | $ 1,587 | 2660 | 2722 | $ 4,221,742 | $ 4,320,144 |
| 2 | 2004 | $ 1,512 | 6874 | 7081 | $ 10,395,695 | $ 10,708,745 |
| 2 | 2005 | $ 1,585 | 6407 | 6559 | $ 10,156,500 | $ 10,397,453 |
| 2 | 2006 | $ 1,555 | 6422 | 6553 | $ 9,989,243 | $ 10,193,010 |
| 3 | 2002 | $ 2,048 | 50877 | 50883 | $ 104,190,543 | $ 104,202,831 |
| 3 | 2003 | $ 2,047 | 14083 | 14181 | $ 28,829,380 | $ 29,029,997 |
| 3 | 2004 | $ 2,091 | 38981 | 39480 | $ 81,506,473 | $ 82,549,846 |
| 3 | 2005 | $ 2,007 | 36636 | 36926 | $ 73,524,080 | $ 74,106,075 |
| 3 | 2006 | $ 1,886 | 31265 | 31473 | $ 58,971,208 | $ 59,363,532 |
